# Monocular metasurface camera for passive single-shot 4D imaging

**DOI:** 10.1038/s41467-023-36812-6

**Published:** 2023-02-23

**Authors:** Zicheng Shen, Feng Zhao, Chunqi Jin, Shuai Wang, Liangcai Cao, Yuanmu Yang

**Affiliations:** State Key Laboratory of Precision Measurement Technology and Instruments, Department of Precision Instrument, Tsinghua University, Beijing, 100084 China

**Keywords:** Metamaterials, Nanophotonics and plasmonics, Imaging and sensing

## Abstract

It is a grand challenge for an imaging system to simultaneously obtain multi-dimensional light field information, such as depth and polarization, of a scene for the accurate perception of the physical world. However, such a task would conventionally require bulky optical components, time-domain multiplexing, and active laser illumination. Here, we experimentally demonstrate a compact monocular camera equipped with a single-layer metalens that can capture a 4D image, including 2D all-in-focus intensity, depth, and polarization of a target scene in a single shot under ambient illumination conditions. The metalens is optimized to have a conjugate pair of polarization-decoupled rotating single-helix point-spread functions that are strongly dependent on the depth of the target object. Combined with a straightforward, physically interpretable image retrieval algorithm, the camera can simultaneously perform high-accuracy depth sensing and high-fidelity polarization imaging over an extended depth of field for both static and dynamic scenes in both indoor and outdoor environments. Such a compact multi-dimensional imaging system could enable new applications in diverse areas ranging from machine vision to microscopy.

## Abstract (web summary)

The authors present a monocular camera equipped with a single-layer metalens for passive single-shot 4D imaging. It can simultaneously perform high-accuracy depth sensing and highfidelity polarization imaging over an extended depth of field.

## Introduction

Conventional cameras can only capture 2D images. In recent years, there has been a rapid development in 3D imaging techniques^1–3^ towards emerging applications such as consumer electronics and autonomous driving. Moreover, a camera that can capture extra dimensions of the light field information, such as polarization and spectrum, may reveal even richer characteristics of a scene^4–6^, thus allowing the perception of a more “complete” physical world towards various tasks, such as object tracking and identification, with high precision.

However, capturing light field information beyond 2D intensity typically requires an optical system with a considerably increased size, weight, and power consumption. For instance, 3D imaging systems based on structured light^7^ and time-of-flight^8^ require active laser illumination. Binocular or multi-view cameras have a large form factor and a depth estimation accuracy and range constrained by their baseline length^9^. Polarization imaging systems are often based on amplitude or focal plane division or require time-domain multiplexing^10^. The simultaneous measurement of multi-dimensional light field information can be even more challenging, often requiring an imaging system with a form factor and complexity far surpassing conventional cameras^11–13^.

It is highly desirable to have a compact monocular camera that can capture multi-dimensional light field information in a single shot under ambient illumination conditions. According to a generalized multi-dimensional image formation model^13^,

1 $$\begin{array}{c}g\left({x}^{{\prime} },{y}^{{\prime} }\right)={\int }_{-\infty }^{\infty }{\int }_{-\infty }^{\infty }\,{f}_{z,p,\ldots }(x,\,y)\,{{{\mbox{PSF}}}}_{z,p,\ldots }({x}^{{\prime} }-x,{y}^{{\prime} }-y)\,{{\mbox{d}}}x{{\mbox{d}}}y\,+\,\eta,\end{array}$$

where (*x*, *y*, *z*) and (*x*’, *y*’) represent the spatial coordinate in the object space and on the image plane, respectively. *f* is the system’s input (target object) that contains multi-dimensional light field information, *g* is the system’s output, *p* is the polarization state of light reflected from the target object, and *η* is a generic noise term. PSF is the point-spread function, which describes the imaging system’s impulse response to a point light source. To obtain light field information beyond the 2D projection of intensity, such as depth *z* and polarization *p* of the target object, the PSF of the system should be strongly dependent on *z* and *p*. This degree of dependency is quantified by Fisher information^14,15^ which determines the estimation accuracy of the corresponding parameter with a given system noise.

Taking depth estimation as an example, since any standard lens has a defocus that is dependent on the depth of the object, monocular cameras have already been used to estimate the depth of a scene by capturing multiple images under different defocus settings^16–18^. However, the depth-from-defocus method typically requires the physical movement of the imaging system and suffers from a low estimation accuracy due to the self-similarity of the system’s PSF along the depth dimension.

More sophisticated depth-dependent PSFs, such as the double-helix PSF, have been proposed for depth estimation with a higher accuracy^14,15,19,20^. The double-helix PSF has two foci rotating around a central point, with the rotation angle dependent on the axial depth of the target object. A double-helix PSF can be generated using diffractive optical elements with a stringently tailored phase profile^14,20^. Subsequently, the depth of the target object can be retrieved by analyzing the power cepstrum of the acquired image^20^. However, the retrieval algorithm is computationally demanding and slow. Furthermore, due to the superposition of the twin-image generated by the two foci, the double-helix PSF method requires an additional reference image with an extended depth of field in order to reconstruct a high-fidelity 2D all-in-focus image of the scene. The reference image can be generated by an additional aperture or time-domain multiplexing but at the cost of significantly increasing the complexity of the imaging system^21^. Moreover, due to the *C*_2_ symmetry of the double-helix PSF, its depth measurement range is limited by the maximum rotation angle of 180°.

To construct a monocular camera that can efficiently retrieve multi-dimensional light field information of a scene in a single shot is an even more challenging task. Recently, an emerging class of subwavelength diffractive optical element, metasurface^22–28^ has been found to be highly versatile to tailor the vectorial light field. Consequently, it opens up new avenues for various applications, including depth^29–32^, polarization^33–36^, and spectral^37,38^ imaging. Very recently, Lin et al. theoretically proposed to leverage the end-to-end optimization of a meta-optics frontend and an image-processing backend for single-shot imaging over a few discrete depth, spectrum, and polarization channels^39^. It remains a major challenge to build a compact multi-dimensional imaging system that allows high-accuracy depth sensing for arbitrary depth values in a wide range of indoor and outdoor scenes.

In this work, we experimentally demonstrate a monocular camera equipped with a single-layer metasurface that can capture 4D light field information, including 2D all-in-focus intensity, depth, and polarization, of a target scene in a single shot. Leveraging the versatility of metasurface to manipulate the vectorial field of light, we design and optimize a polarization-multiplexed metasurface with a decoupled pair of conjugate single-helix PSFs, forming a pair of spatially-separated twin-image of orthogonal polarization on the photosensor. The depth and polarization information of the target scene is simultaneously encoded in the decoupled twin-image pair. The PSF of the metasurface has a Fisher information two orders of magnitude higher than that of a standard lens. Combined with a straightforward image retrieval algorithm, we demonstrate high-accuracy depth estimation and high-fidelity polarization imaging over an extended depth of field for both static and dynamic scenes under ambient lighting conditions.

## Results

### Single-shot 4D imaging framework

**Fig. 1:**
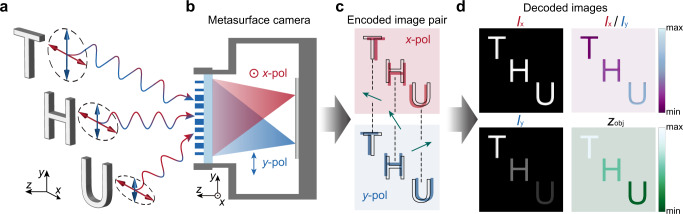
Framework of using a monocular metasurface camera for single-shot 4D imaging. **a** The target scene consists of objects with different polarization states and depths. **b** Light emitted from the scene is split and focused on the photosensor, forming two images of orthogonal linear polarizations. The red and blue colors correspond to *x*- and *y*-polarized light, respectively. **c** The raw encoded image-pair recorded on the photosensor, with depth information of the scene encoded in the local orientation of the translation vector (green arrows). The black outlines are the geometric image positions of the objects. **d** Decoded 4D image of the target scene, including all-in-focus 2D light intensity (*I*_x_ and *I*_y_), polarization contrast (*I*_x_/*I*_y_), and depth (*z*_obj_), can be retrieved from the raw image-pair by a straightforward, physically interpretable algorithm based on image segmentation and template matching of the object-pair.

The framework of the monocular metasurface camera for single-shot 4D imaging is schematically illustrated in Fig. 1. A single-layer metasurface is designed and optimized to generate a pair of conjugate single-helix PSFs that form a twin-image pair of the target object with orthogonal linear polarization laterally shifted on the photosensor (Fig. 1a). The depth of the scene is encoded in the local orientations of the translation vectors of the twin-image (Fig. 1b, c). Subsequently, one can computationally retrieve the all-in-focus 2D light intensity, depth, and polarization contrast of the scene (Fig. 1c, d).

### Metasurface design and characterization

**Fig. 2:**
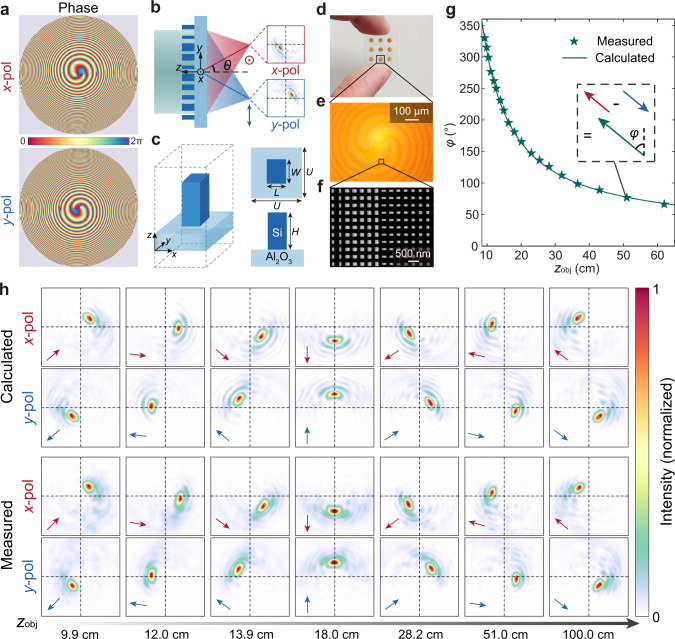
Metasurface design and characterization. **a** Optimized metasurface phase profiles that can generate a decoupled pair of conjugate single-helix PSFs for *x*-polarized and *y*-polarized incident light, respectively. **b** Schematic of the metasurface that splits and focuses the light of orthogonal polarization on laterally shifted location of the photosensor with a decoupled pair of conjugate single-helix PSFs, where *θ* = 8° is the off-axis angle for polarization splitting. **c** The unit-cell of the metasurface is composed of silicon nanopillars of rectangular in-plane cross-sections on a sapphire substrate, with height *H* = 600 nm, period *U* = 350 nm, and width *W* and length *L* varying between 100 nm and 250 nm. **d**–**f** Photograph (**d**), optical microscopy image (**e**), and scanning electron microscopy image (**f**) of the fabricated metasurface sample, respectively. The other metalenses as shown in the photograph are fabricated for different purposes. **g** Calculated (green line) and measured (green star) orientation angle *φ* of the single-helix PSF pair as a function of the axial depth of a point object *z*_obj_. The inset shows the method to extract *φ* from the translation relationship of the decoupled pair of conjugate single-helix PSFs. **h** Calculated and experimentally measured single-helix PSFs as a function of *z*_obj_, for *x*-polarized and *y*-polarized incident light, respectively. The PSF pairs are conjugate with respect to the geometric image point (intersection point of black crosshairs). The arrows represent the vectors pointing from the geometric image point to the maximum of the PSFs.

To generate a rotating single-helix PSF at a near-infrared operation wavelength *λ* = 800 nm, we assume the placement of a metasurface at the entrance pupil of the imaging system, and initialize the transmission phase of the metasurface with Fresnel zones carrying spiral phase profiles with gradually increasing topological quantum numbers towards outer rings of the zone plate^40,41^ as,

2 $$\,{\psi }_{r}\left(u,\, {\varphi }_{u}\right)=\left\{l{\varphi }_{u}{{{{{\rm{|}}}}}}{\left(\frac{l-1}{L}\right)}^{\varepsilon }\le u\le {\left(\frac{l}{L}\right)}^{\varepsilon },\, l=1,\, \ldots,\, L\right\},$$

where *u* is the normalized radial coordinate and ${\varphi }_{u}$ is the azimuth angle in the entrance pupil plane. $[L,\,\varepsilon ]$ are adjustable design parameters. Compared with Gauss–Laguerre mode-based approach widely used in the design of double-helix PSFs^30,42,43^, the adopted Fresnel zone approach can generate a more compact rotating PSF with the shape of the PSF kept almost invariant over an extended depth of field^40^. Subsequently, an iterative Fourier transform algorithm is used to maximize the energy in the main lobe of the rotating PSF within the 360° rotation range. The iterative optimization process further improves the peak intensity of the main lobe of the PSF by 36%. In the final step of designing the transmission phase profile, a polarization splitting phase term,

3 $${\psi }_{{xf}}\left(x,y\right)=-\frac{2\pi }{\lambda }\left[\sqrt{{x}^{2}+{y}^{2}+{f}^{2}+2{xf}{{\sin }}\theta }-f\right],$$

4 $${\psi }_{{yf}}\left(x,y\right)=-\frac{2\pi }{\lambda }\left[\sqrt{{x}^{2}+{y}^{2}+{f}^{2}-2{xf}{{\sin }}\theta }-f\right],$$

is added to spatially decouple the conjugate single-helix PSFs, where *f* = 20 mm is the focal length, *θ* = 8° is the off-axis angle for polarization splitting. The optimized phase profiles for both *x*- and *y*-polarized incident light are shown in Fig. 2a, along with the schematic of the metasurface that splits and focuses the light of orthogonal polarization, as shown in Fig. 2b. More detailed discussion of the transmission phase design is included in Supplementary Section 1.

The unit-cell of the metasurface is composed of silicon nanopillars of rectangular in-plane cross-sections (Fig. 2c), thus allowing the independent control of the phase of *x*- and *y*-polarized incident light over a full 2π range with near-unity transmittance (Supplementary Section 2). The smallest gaps between nanopillars are designed to be over 100 nm to reduce coupling between nanopillars^44,45^. The metasurface is fabricated using a complementary metal-oxide-semiconductor (CMOS)-compatible process (“Methods” and Supplementary Section 2), with an aperture diameter of 2 mm. The photograph, optical microscopy image, and scanning electron microscopy image of the fabricated metasurface are shown in Fig. 2d–f, respectively. We measure the polarization-dependent PSF pairs of the fabricated metasurface as a function of the axial depth of a point object (*z*_obj_) (“Methods” and Supplementary Section 3) and confirm it is in close agreement with the calculation (Fig. 2g, h). The lens is measured to have a polarization extinction ratio of 35.6 (Supplementary Section 3) and a diffraction efficiency^46^ of 44.54% and 43.93% for each input linear polarization (“Methods” and Supplementary Section 4). Since the single-helix PSF has a full 360° rotation range, its depth measurement range can be significantly extended compared to imaging systems with a double-helix PSF.

### 4D imaging experiments

**Fig. 3:**
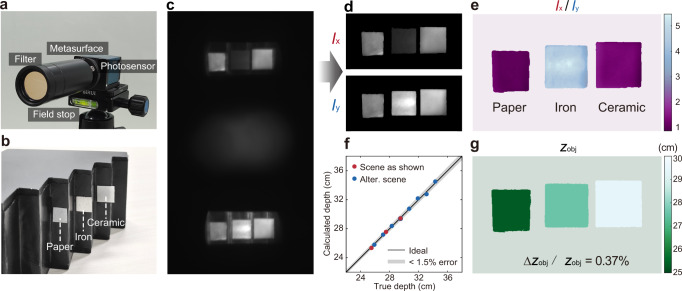
Indoor imaging of a static scene. **a** Photograph of the metasurface camera. **b** Photograph of the target scene consisting of three pieces of different materials (paper, iron, and ceramic) located at different axial depths. **c** Raw image-pair captured by the metasurface camera. **d** Decoded all-in-focus polarization image pair *I*_*x*_ and *I*_*y*_. **e** Polarization contrast of the scene, defined as *I*_*x*_/*I*_*y*_, which clearly distinct metallic and non-metallic materials. **f** Comparison of the calculated depth to the ground truth for the scene shown here and an alternative scene as shown in Supplementary Section 5. **g** Retrieved depth map of the scene, with a normalized mean absolute error (Δ*z*_obj_/*z*_obj_) of only 0.37%.

We assemble the metasurface with a CMOS-based photosensor with an active area of 12.8 × 12.8 mm^2^, as shown in Fig. 3a. A bandpass filter with a central wavelength of 800 nm and a bandwidth of 10 nm and an aperture are installed in front of the camera to limit the spectral bandwidth and field of view (FOV), respectively. The assembled camera system has a size of 3.1 × 3.6 × 13.5 cm^3^, which may be further reduced with a customized photosensor and housing. To demonstrate single-shot 4D imaging, we set up a scene consisting of three pieces of different materials (paper, iron, and ceramic) located at different axial depths, as shown in Fig. 3b. When a partially polarized near-infrared light-emitting diode illuminates the scene, a raw image with depth and polarization information of the scene encoded can be captured (Fig. 3c).

The metasurface camera with a polarization-decoupled pair of conjugate single-helix PSFs forms two spatially separated images on the photosensor. Consequently, it avoids the superposition of twin-image presented in imaging systems with double-helix PSF. This allows high-fidelity all-in-focus 2D light intensity, depth, and polarization retrieval of the target scene using a straightforward, physically interpretable algorithm based on image segmentation and calculation of the local orientation of the translation vector of the object pair (Supplementary Section 5). It takes less than 0.4 s to reconstruct 4D images, each with up to 500 × 1000 pixels, from a raw measurement on a laptop computer with Intel i7-10875H CPU and 16 GB RAM. This computation speed can be universally applied to 4D image reconstruction of any given scenes.

Figure 3d shows the retrieved all-in-focus 2D intensity images of the target scene for *x*- and *y*-polarized light, denoted as *I*_*x*_ and *I*_*y*_, respectively. The polarization contrast can be calculated subsequently as *I*_*x*_/*I*_*y*_, which facilitates the clear distinction of metallic and non-metallic materials in the target scene (Fig. 3e). In comparison with the true depth, the retrieved depth map has a modest normalized mean absolute error (NMAE) value of 0.37%, defined as the mean value of the absolute error divided by its ground truth (Fig. 3f, g). We further verify that the NMAE of depth estimation can be below 1% for an alternative scene with more objects over depths ranging from 25 cm to 34 cm (Supplementary Section 5).

**Fig. 4:**
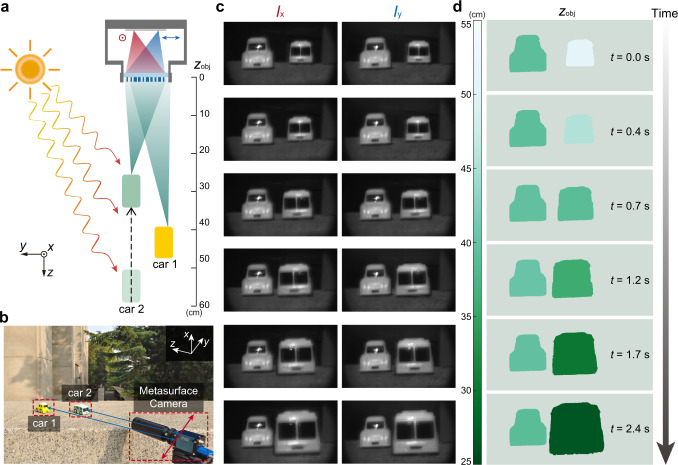
Outdoor imaging of a dynamic scene. **a**, **b** Schematic (**a**) and photograph (**b**) of the outdoor scene for dynamic imaging experiment under sunlight illumination. Car 2 moves towards the metasurface camera at a nonuniform speed of ~10 cm/s, while car 1 stays still. **c** Decoded all-in-focus polarization image pairs *I*_*x*_ and *I*_*y*_ of the scene for selected frames of the recorded video. **d** Retrieved depth maps of the scene for selected frames of the recorded video.

A monocular camera is capable of capturing depth and intensity images for a dynamic scene under ambient lighting conditions in a single shot. To validate the concept, we record a video of a dynamic scene of moving toy cars (one toy car is kept still, the other moves at a nonuniform speed of ~10 cm/s) using the monocular metasurface camera under sunlight illumination. The schematic and photograph of the scene are shown in Fig. 4a, b, respectively. Figures 4c and 4d show raw image pairs captured by the metasurface camera along with retrieved depth maps for selected frames of the recorded video (Supplementary Movie 1, played at 0.3× speed), which clearly reveals the absolute depth values and space-time relationship of the dynamic 3D scene with one of the toy cars moving over a distance of about 25 cm. The NMAE of depth estimation for the still and the moving toy car are 0.78% and 1.26%, respectively (Supplementary Section 5). The slightly higher depth estimation error for the outdoor dynamic scene, in comparison with indoor static scenes, may be due to the longer depth range, as well as the trade-off between signal-to-noise ratio and motion artifact of the captured images. Note that a longer integration time may lead to better signal-to-noise ratio but more image blur.

## Discussion

The prototype metasurface camera shown here can perform high-accuracy depth estimation in the centimeter range. For applications aiming at a further distance, one can scale up the lens aperture. The depth estimation accuracy of the metasurface camera at a certain range is proportional to the rotation speed of the single-helix PSF, which is proportional to the square of the aperture size. For instance, an aperture size of 5 cm may allow depth estimation at 200-m-range with an estimated mean depth error still well below 1% (Supplementary Section 6).

The metasurface camera with the conjugate single-helix PSF has a Fisher information along the depth dimension over two orders of magnitude higher than that of a standard lens, resulting in much higher depth estimation accuracy (Supplementary Section 7). Furthermore, the single-helix PSF also allows the direct acquisition of high-quality 2D images with an extended depth of field, without the need for additional reference images (Supplementary Section 8).

The prototype metasurface camera works over a narrow spectral band and FOV. However, based on the multi-dimensional imaging framework, one may leverage the chromatic dispersion of a more sophisticated metasurface design to realize PSFs that are also strongly dependent on the wavelength of the incident light. In such a way, one may also achieve multispectral or even hyperspectral imaging using a monochromatic sensor. Using multiplexed meta-atoms, one may also achieve full-Stokes polarization imaging by directing the light of 3 pairs of orthogonal polarization states onto different areas of the photosensor plane (Supplementary Section 9). With the additional spectral or polarization channels, an important question is how to maximize the total channel capacity without greatly sacrificing the spatial resolution or FOV of the imaging system, given the finite pixel number of the photosensor. Such a task may be partially fulfilled by compressive sensing^47^. To further increase the off-axis angle and the FOV of the camera system, one could use multi-level diffractive optics^48^, combine multiple layers of metasurfaces^49,50^, or by using proper aperture stops^51^. Although we only demonstrated depth estimation for segmented objects with uniform depth values here, ultimately, we expect that the complement of more advanced image retrieval algorithms, such as deep learning^52,53^ and compressive sensing^47,54^, may facilitate accurate pixel-wise multi-dimensional image rendering for complex scenes. It is worth noticing that although deep learning has been proven to be a potent tool for numerous imaging tasks, including monocular depth estimation^55–59^, the method presented here is physics-driven and fully interpretable, without its operation relying on a prescribed training dataset that often has a bias towards certain scenario.

In summary, we have demonstrated a metasurface camera capable of capturing a high-fidelity 4D image, including 2D intensity, depth, and polarization in a single shot. It exploits the unprecedented ability of metasurface to manipulate the vectorial light field to allow multi-dimensional imaging using a single-piece planar optical component, with performance unattainable with conventional refractive or diffractive optics. A compact monocular camera system for multi-dimensional imaging may be useful in a myriad of application areas, including but not limited to augmented reality, robot vision, autonomous driving, remote sensing, and biomedical imaging.

## Methods

### Metasurface fabrication

The fabrication of the metasurface is done through a commercial service (Tianjin H-chip Technology Group). The process is schematically shown in Supplementary Fig. S6. The fabrication starts with a silicon-on-sapphire substrate with a thickness of the monocrystalline silicon film of 600 nm. Electron beam lithography (JEOL-6300FS) is employed to write the metasurface pattern using a negative tone resist Hydrogen silsesquioxane (HSQ). Next, the pattern is transferred to the silicon layer via dry etching directly using HSQ as the mask. Finally, the HSQ resist is removed by buffered oxide etchant.

### PSF and diffraction efficiency measurement setup

To measure the PSF of the fabricated metasurface, we construct a setup as shown in Supplementary Fig. S8. The illumination source consists of collimated light from a supercontinuum laser (YSL SC-PRO-15) and a bandpass filter (Thorlabs FB800-10) with a central wavelength of 800 nm and a bandwidth of 10 nm. The laser beam is expanded by a beam expander and focused by a convex lens with a focal length of 35 mm to generate the point light source. When measuring the PSF of the metasurface, the distance between the point light source and the metasurface is varied, with the distance between the metasurface and the photosensor kept fixed.

To estimate the polarization-dependent diffraction efficiency of the metasurface, the collimated laser beam is filtered by a linear polarizer (Thorlabs LPNIR100-MP2), with its spot size reduced using a convex lens with a focal length of 150 mm to match the aperture size of the metasurface. An optical power meter (Thorlabs PM122D) is first placed in front of the metasurface to measure the power of the incident light ${{P}}_{{{\mbox{inc}}}}$. Subsequently, to measure the power of the focused light ${P}_{{{\mbox{f}}}},$ a pinhole of 100-μm diameter is placed in front of the power meter. The position of the power meter is spatially scanned and maximized near the designed focal point of the metasurface. The diffraction efficiency of the metasurface is estimated as $\eta \,={P}_{{{\mbox{f}}}}\,/{P}_{{{\mbox{inc}}}}$.

## Supplementary information

Supplementary Information

Description of Additional Supplementary Files

Supplementary Movie 1

## Data Availability

The data that support the plots within this paper and other findings of this study are available from the corresponding author on request.

## Code availability

The code that support the plots within this paper and other findings of this study are available from the corresponding author on request.
