# Supplementary material for: Monocular metasurface camera for passive single-shot 4D imaging
Source: Nat Commun. 2023 Feb 23;14:1035. doi: 10.1038/s41467-023-36812-6 (PMC9950364; doi:10.1038/s41467-023-36812-6)
Supplement: Supplementary file 1 — Supplementary Information [file 41467_2023_36812_MOESM1_ESM.pdf]

Supplementary Information:

Monocular metasurface camera for passive single-shot 4D imaging

Zicheng Shen, Feng Zhao, Chunqi Jin, Shuai Wang, Liangcai Cao, Yuanmu Yang*

State Key Laboratory of Precision Measurement Technology and Instruments, Department of Precision Instrument, Tsinghua University, Beijing 100084, China

*Corresponding author: ymyang@tsinghua.edu.cn

1. Single-helix point spread function (PSF) design and optimization

The general solution of a shape-invariant rotating beam was first derived from the Gauss-Laguerre function[1,2]. To generate a rotating PSF, one can, conventionally, design a diffractive optical element with the phase profile generated from the superposition of Gauss-Laguerre modes[3-5]. Here, we use an alternative Fresnel zone approach to design the phase profile that generates the single-helix PSF. The Fresnel zone approach[6,7] arranges phase term with ring areas carrying spiral phase profiles of increasing topological quantum numbers toward outer rings, with an additional focusing term, in the entrance pupil of the imaging system. Compared with Gauss-Laguerre mode-based approach, the Fresnel zone approach can generate a more compact rotating PSF with the shape of the PSF remaining almost invariant over a large depth range[7]. The Fresnel zone phase term $\psi_r(u, \phi_u)$ for generating a single-helix PSF is given by:

$$\psi_r(u, \varphi_u) = \left\{ l\varphi_u \middle| \left(\frac{l-1}{L}\right)^{\varepsilon} \leq u \leq \left(\frac{l}{L}\right)^{\varepsilon}, l = 1, \ldots, L \right\} \quad \text{(S1)}$$

where u is the normalized radial coordinate and φ_u is the azimuth angle in the entrance pupil plane. $[L, \varepsilon]$ are adjustable design parameters. When $L \gg 1$ and $\varepsilon = 0.5$, with the addition of a focusing phase term, we can calculate the complex amplitude of the PSF based on the Fresnel integral as[6]:

$$U(r, \varphi; \zeta) \approx 2\sqrt{\pi} \exp[-i\zeta/(2L)] \frac{\sin[\zeta/(2L)]}{\zeta} \times \sum_{l=1}^{L} i^l \exp[-il(\varphi - \zeta/L)] J_l\left(\frac{2\pi\sqrt{l/L}r}{r_0}\right) \quad \text{(S2)}$$

where r is the normalized radial coordinate and φ is the azimuthal angle with respect to the geometric image point, which refers to the centre of the PSF when the Fresnel zone phase term is not added. r_0 is the radius of the in-focus diffraction spot. ζ is the defocus parameter given by:

$$\zeta = \frac{\pi}{\lambda}\left(\frac{1}{z_{\text{obj}}} - \frac{1}{z_f}\right) R^2 \quad \text{(S3)}$$

where R is the radius of the entrance pupil, z_{obj} is the actual object depth, and z_f is the depth of the in-focus object plane.

According to Eq. S2, when $\zeta << 2\pi L$, the complex amplitude of PSF is related to ζ via the term $\exp[-il(\phi - \zeta/L)]$. Therefore, both the complex amplitude and the intensity of the

PSF, $PSF(r,\phi;\ \zeta) = |U(r,\phi;\ \zeta)|^2$, remain shape- and size-invariant, and rotates at the speed of $1/L$ rad per unit of ζ. The defocus parameter ζ relates the object depth to the rotation angle of the PSF. For the parameters $[L,\varepsilon]$, L can be adjusted to control the rotation speed, while ε can be tuned to balance the trade-off between the main-lobe confinement and the shape-invariance during the rotation of the PSF.

Considering the prototype imaging system with $R = 1$ mm, $f = 20$ mm, and $\lambda = 800$ nm, we fine-tune and set $[L,\varepsilon] = [8, 0.8]$. Additional focusing and polarization-splitting terms are subsequently added. The full phase profile of the metasurface that has a polarization-decoupled pair of conjugate rotating PSFs is given by:

$$\psi_x = \psi_{xr} + \psi_{xf} \quad \text{(S4)}$$

$$\psi_y = \psi_{yr} + \psi_{yf} \quad \text{(S5)}$$

where ψ_x and ψ_y are the transmission phase for *x*- and *y*-polarized light, respectively. ψ_{xr} and ψ_{yr} are the Fresnel zone phase terms for generating rotating PSFs (ψ_{yr} is obtained by rotating ψ_{xr} by 180°). ψ_{xf} and ψ_{yf} are the focusing and polarization-splitting terms given by:

$$\psi_{xf}(x,y) = -\frac{2\pi}{\lambda}\left[\sqrt{x^2+y^2+f^2+2xf\sin\theta} - f\right] \quad \text{(S6)}$$

$$\psi_{yf}(x,y) = -\frac{2\pi}{\lambda}\left[\sqrt{x^2+y^2+f^2-2xf\sin\theta} - f\right] \quad \text{(S7)}$$

where $\theta = 8°$ is the off-axis angle for polarization-splitting. Initial Fresnel zone phase terms (ψ_{xr} and ψ_{yr}) and the numerically calculated PSFs as a function of the axis depth of the point light source are shown in **Fig. S1**. We calculate the far-field distributions for *x*- and *y*-polarized incident light, respectively, using the angular spectrum method[8], since it is not limited by the paraxial approximation.

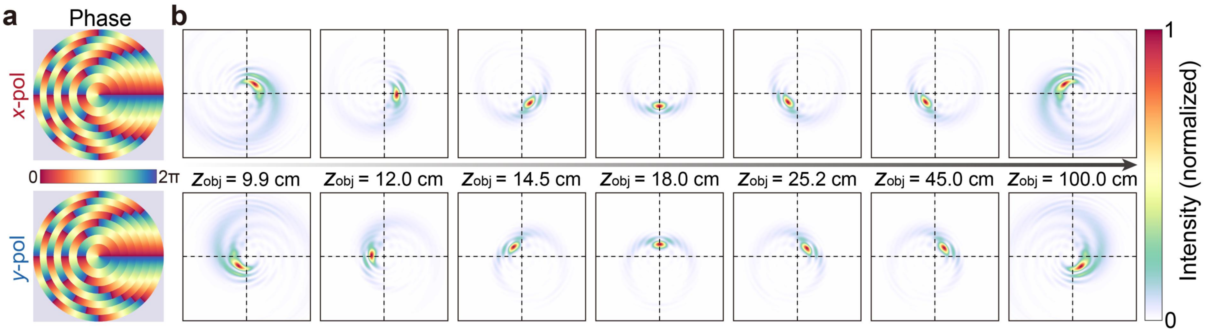

Figure S1 | a, Initial Fresnel zone phase term ψ_{xr} and ψ_{yr} of the metasurface for generating a decoupled pair of conjugate single-helix PSFs. **b,** Calculated single-helix PSFs for *x*-polarized and *y*-polarized incident light, respectively. The PSF pairs are conjugate with respect to the geometric image point (intersection point of crosshairs).

To further improve the quality of the single-helix PSFs generated by the metasurface, thus improving the accuracy of the depth estimation as well as the quality of 2D intensity images, we use an iterative optimization algorithm, with the initial Fresnel zone phase term design as the input, to maximize the energy in the main-lobe of the rotating PSF. The iterative optimization algorithm is schematically shown in **Fig. S2**.

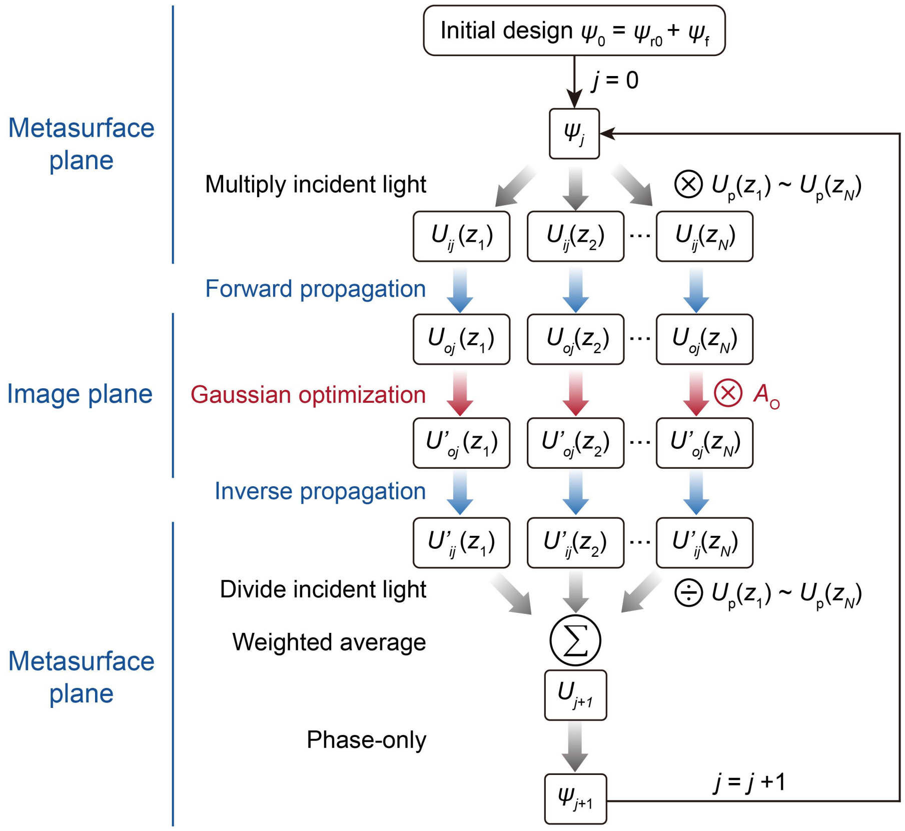

Figure S2 | A flow chart of the iterative Fourier transform algorithm for optimizing the Rotating PSFs of the metasurface, where $U = A\exp(i\psi)$ represents complex amplitude. The forward propagation and inverse propagation are calculated using the angular spectrum method. $U_p(z_1) \sim U_p(z_N)$ are the complex amplitude of point light sources at different axial depths $(z_1 \sim z_N)$ propagated to the metasurface plane.

In this iterative process, we optimize the PSFs within the 360° rotation range, which corresponds to the effective depth estimation range. Starting from the initially designed phase profile of the metasurface ψ_0, we calculate complex amplitudes of PSFs corresponding to 9 different on-axis point light sources ($N = 9$) with depths between 9 cm and 100 cm, corresponding to $U_{oj}\,(z_1)$ to $U_{oj}\,(z_9)$ in Fig. S2. A_O is a two-dimensional Gaussian function centred at the peak of the main lobe of the PSF with cut-off boundaries, which is used to iteratively increase the fraction of light energy confined in the main lobe of the single-helix PSF. The optimized PSFs $U'_{oj}\,(z_1)$ to $U'_{oj}\,(z_9)$ are subsequently inversely propagated to the metasurface plane and are weight-averaged after dividing the complex amplitude of the incident light. The resultant complex amplitude is given by:

$$U_{j+1} = \sum_{n=1}^{N} w_n \frac{U_{ij}(z_n)}{U_p(z_n)} \quad \text{(S8)}$$

The weights w_n satisfy $\sum_{n=1}^{N} w_n = 1$. We fine-tune and set w_1 and w_9 to be relatively large to obtain a more uniform image quality throughout the depth estimation range since PSFs corresponding to z_1 and z_9 have relatively low qualities in the initial design. The amplitude of the complex amplitude is subsequently set to unity with the phase retained for the next iteration.

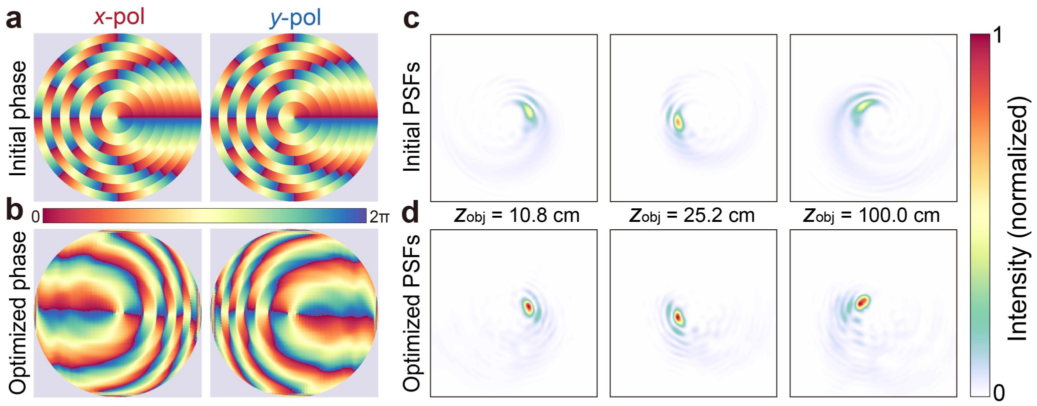

Figure S3 | a, Initial Fresnel zone phase term ψ_{xr} and ψ_{yr} of the metasurface. **b,** Optimized Fresnel zone phase term ψ_{xr} and ψ_{yr} of the metasurface. **c-d,** Comparison between initial and optimized PSFs.

This optimization process is performed for both *x*- and *y*-polarized incident light with ten iterations each. The initially designed and optimized Fresnel zone phase terms of the metasurface for both polarization and corresponding PSFs for *x*-polarized light are shown in **Fig. S3**. The peak intensity and contrast between the main-lobe and the side-lobe of single-helix PSFs are improved by 36% and 71% on average, respectively, within the 360° rotation range. The full optimized phase profile of the metasurface incorporating the additional focusing and polarization-splitting term (ψ_x and ψ_y) and corresponding PSFs for both polarizations are shown in **Fig. 2** of the main text.

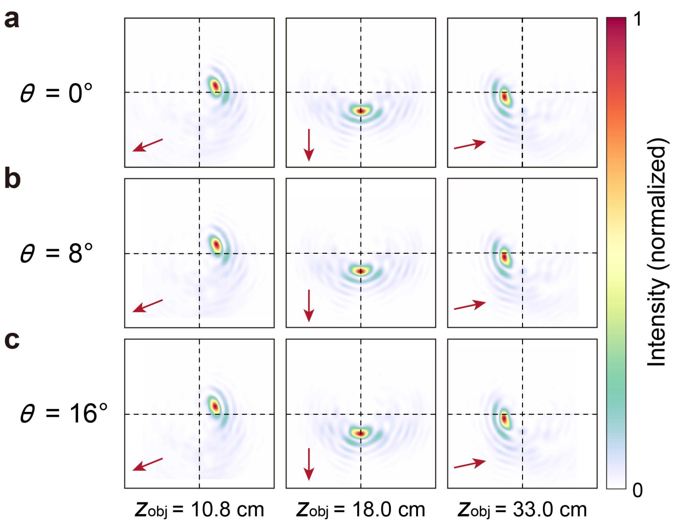

Figure S4 | Calculated single-helix PSFs generated by the optimized phase design, for *y*-polarized incident light, when off-axis angle $\theta = 0°$ (**a**), 8° (**b**) and 16° (**c**), respectively. All calculations are done with the in-focus object plane placed at $z_{obj} = 18.0$ cm.

We further evaluate the effect of off-axis angle (θ) on the quality of the single-helix PSF using a far-field calculation based on the angular spectrum method. The results show that the off-axis angle of 8° and 16° has little effect on the rotation angle and the shape of the single-helix PSF, as shown in **Fig. S4**. Note that with the off-axis angle induced by the polarization-splitting phase term, the PSF shown in Fig. S4 is still on-axis.

2. Metasurface unit-cell design and fabrication

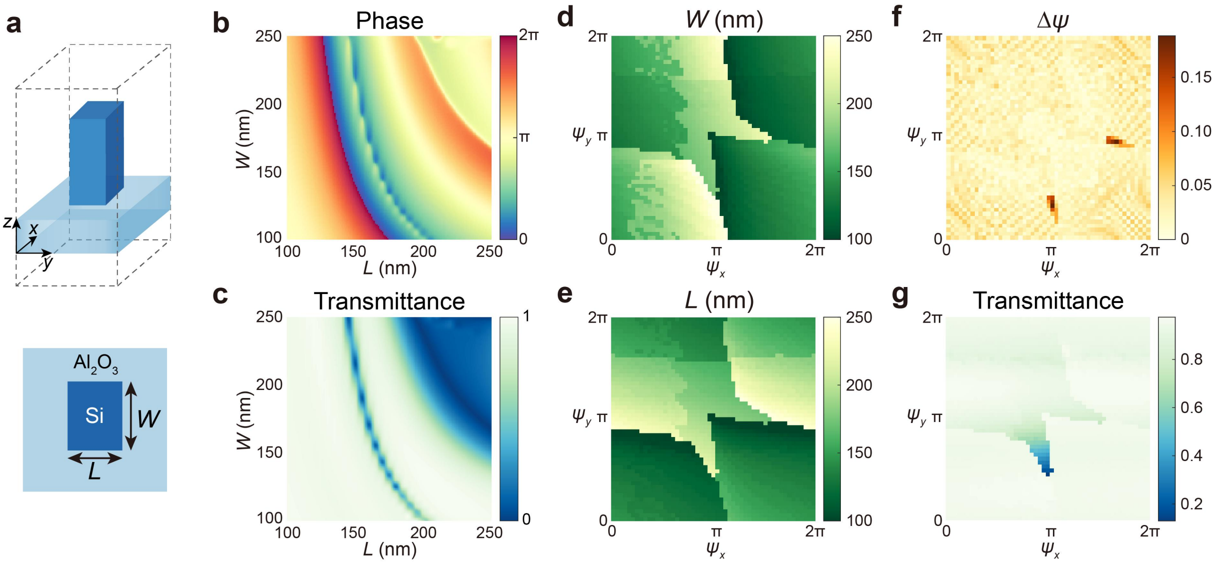

Figure S5 | a, Tilted and top view of the unit cell of the metasurface. **b,** Simulation transmission phase for *x*- polarized normally incident plane wave. **c,** Simulation transmittance for *x*-

polarized normally incident plane wave. **d-e**, Selected length L and width W of the nanopillars for independent and continuous control of the transmission phase of *x*- and *y*-polarized incident light. **f**, Phase error $\Delta\psi$ of selected structures. **g**, Transmittance of selected structures.

The unit-cells of the metasurface are made of 600-nm-tall single-crystalline silicon nanopillar on a 500-μm-thick sapphire substrate. The refractive index of single-crystalline silicon was measured to be 3.717 with minimum loss at λ = 800 nm. We simulate the transmission amplitude and phase of the metasurface as a function of the nanopillar's width and length using the finite-difference time-domain (FDTD) method for *x*- and *y*-polarized incident light, respectively, at λ = 800 nm, assuming a periodic boundary condition, to serve as the library for our metasurface design. The width W and length L of the nanopillar are swept in the range of 100 nm to 250 nm, while the period U and height H are fixed at 350 nm and 600 nm, respectively. The simulation results of phase and transmittance for both *x*-polarized normally incident light are shown in **Fig. S5b,c**, and the phase and transmittance of *y*-polarized light are the transposes of L and W of the response for *x*-polarized light. From the simulation result, a pair of $[L, W]$ is selected for each pair of target transmission phase of *x*- and *y*-polarized incident light to realize independent and continuous phase control, as is shown in **Fig.S5d,e**. The phase error $\Delta\psi$ of selected structures is mostly below 0.05 rad, as is shown in **Fig.S5f**. The transmittance of selected structures is mostly higher than 0.9, as is shown in **Fig.S5g**. The fabrication process of the metasurface is detailed in **methods** and shown in **Fig. S6**.

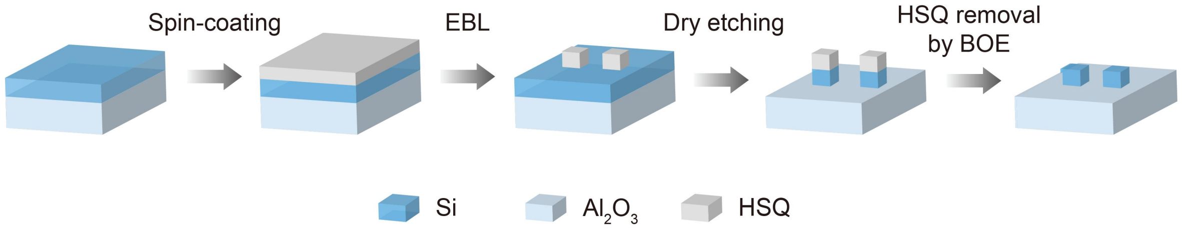

Figure S6 | Schematic of the fabrication process flow chart of the metasurface (EBL: electron-beam-lithography, HSQ: Hydrogen silsesquioxane, BOE: buffered oxide etchant).

3. Characterization of imaging performance of the fabricated metasurface

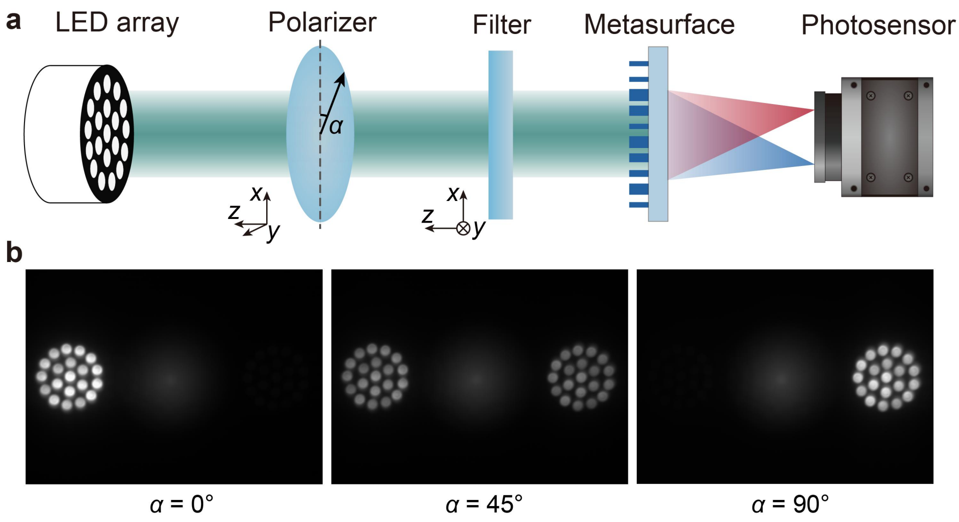

Figure S7 | a, Schematic of the experimental setup for the characterization of the polarization imaging ability of the metasurface. **b,** Images of the LED array taken with the metasurface-based flat lens with different polarization angles α of the linear polarizer.

The experimental setup for characterizing the polarization imaging capability of the metasurface is shown in **Fig. S7**. The target object is a light-emitting diode (LED) array with a central wavelength of 780 nm (Thorlabs LIU780A). The light incident from the LED array is filtered by a bandpass filter with a central wavelength of 800 nm and a bandwidth of 10 nm (Thorlabs FB800-10) and a linear polarizer (Thorlabs LPNIR100-MP2) before impinging on the metasurface. Three images with the polarization angle of the linear polarizer rotating from 0° to 90° are displayed in **Fig. S7b**. When the polarization image pair (I_x and I_y) of the LED array reaches the highest contrast by rotating the polarizer, the polarization extinction ratio of the fabricated metasurface can be estimated, from the mean value of I_x/I_y (background noise is subtracted), to be 35.6.

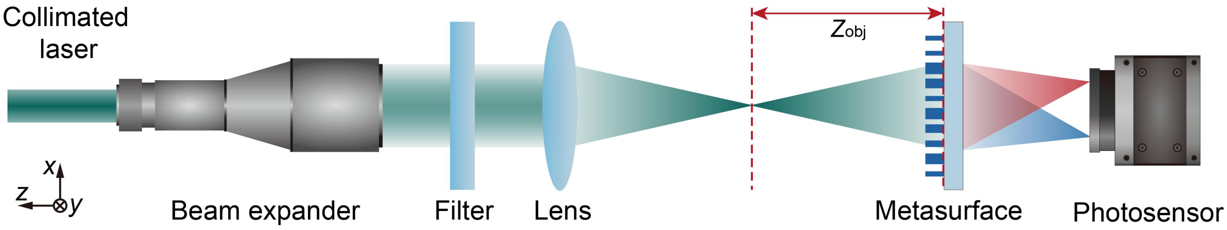

Figure S8 | Schematic of the experimental setup for the measurement of PSFs of the metasurface.

The setup for experimental measurement of PSFs of the metasurface is detailed in **methods** and schematically shown in **Fig. S8**. The light is incident from the nanopillar side of the metasurface in the experimental measurement of PSFs of the metasurface since we find that

the birefringence in the sapphire substrate may cause performance deterioration of the polarization extinction.

4. Diffraction efficiency of the fabricated metasurface

We estimate the diffraction efficiency of the metasurface-based flat lens by performing a simulation of a metasurface with a relatively small aperture (50-μm-diameter) using FDTD since the full simulation of a 2-mm-aperture metasurface requires an enormous amount of computer memory, which is not currently available to us. The simulated diffraction efficiency of *x*- and *y*-polarized incident light are both 54.2%, which are in close agreement with the measured diffraction efficiency of 44.54% and 43.93%. Representative scanning electron microscopy images of the fabricated metasurface are shown in **Fig. S9**.

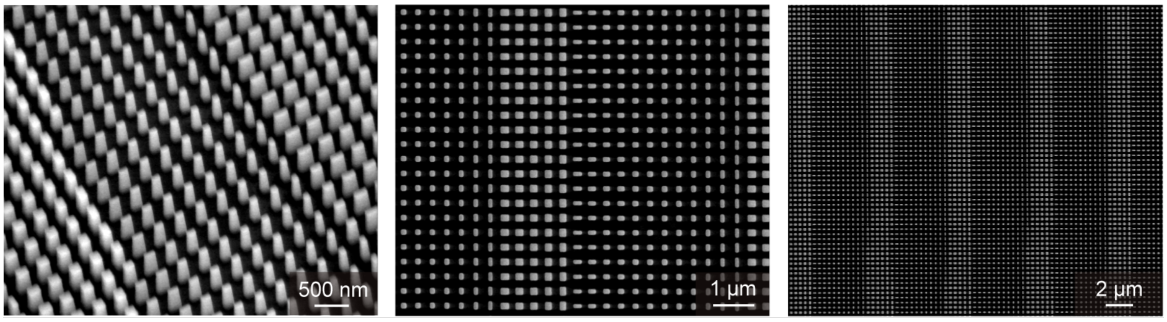

Figure S9 | Scanning electron microscopy images of the fabricated metasurface.

5. Framework of the 4D image retrieval algorithm

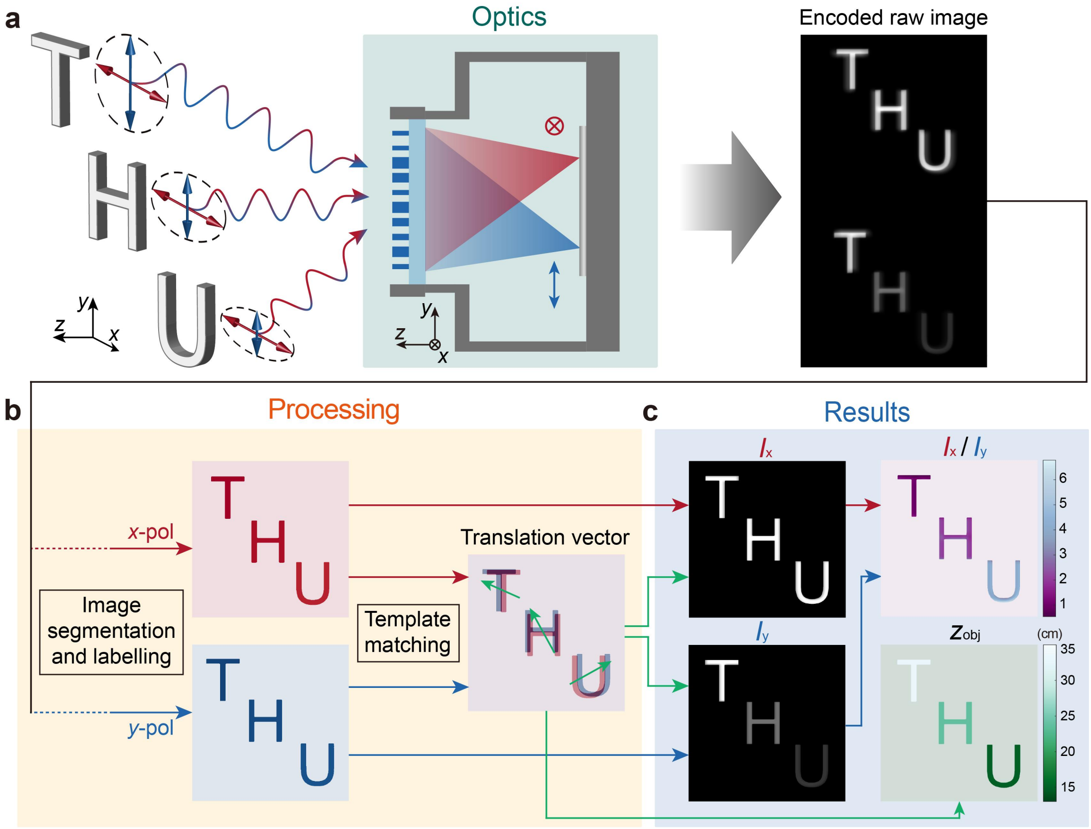

Figure S10 | Framework of the 4D image retrieval algorithm. **a,** the metasurface camera captures a raw image of the scene with depth and polarization information encoded. **b,** Image processing: image pair cut from the encoded raw image in (**a**) is processed by image segmentation and labelling, resulting in a pair of segmented objects with depth information encoded in the translation vector. The translation vector is retrieved from the pair of segmented objects by template matching. **c,** The retrieved 4D light field information, including 2D intensity, depth, and polarization, of the target scene.

As schematically shown in **Fig. S10**, to retrieve multi-dimensional light field information, including 2D intensity, depth, and polarization, of the target scene, we first cut a pair of images from the captured raw image. This pair of images are cut to have identical sizes. Image segmentation is subsequently performed to the image pair to label individual target objects. We first apply Canny edge detector[9] to the encoded image pair. Subsequently, connected area detection is applied to segment objects with closed edges, followed by removing too small objects to avoid noise points and redundant edges. Simple edge detection may result in the segmentation of unwanted areas. For example, for a target with the shape of the letter "A", the enclosed hollow area may also be segmented as a target. Therefore, image binarization with an adaptive threshold is also performed to the encoded image pair. Each labelled area is compared with the result of binarization, and areas with an overlapping ratio less than 0.8 are removed, resulting in an image pair composed of segmented objects.

To calculate the depth of each segmented object, with each segmented object assumed to have a uniform depth, we perform template matching for each segmented object to retrieve the

value of the translation vector. In the template matching process, the template taken from one image slides in the image that is to be matched to search for the best match within the area of possible translation. The goal is to minimize the sum of absolute difference (SAD) of the grayscale value of all pixels combined. Since the modulus of the translation vector is kept almost invariant, the area of possible translation has a ring shape. This helps us minimize the sliding area, thus reducing the computation cost. The depth value is then calculated from the orientation angle of the best-matched translation vector (with minimal SAD value) according to:

$$z(i) = 1/\left[\frac{V+z_f(\varphi(i)-\varphi_f)}{V}z_f\right] \quad \text{(S9)}$$

where $z(i)$ and $\varphi(i)$ are depth and orientation angle of the translation vector for the ith segmented object. z_f and φ_f are the depth of the in-focus object plane and its corresponding orientation angle of the translation vector. V is a constant determined by the initial design of the single-helix PSF.

To retrieve the all-in-focus 2D intensity for both polarization (I_x and I_y), outlines of segmented objects are used to select the area of the target objects from the encoded image, and each object is translated back to the position of the geometric image according to the translation vectors. Subsequently, the polarization contrast (I_x/I_y) can also be retrieved.

To improve the accuracy of depth estimation, the values of the translation vector are refined. Each initially retrieved translation vector is quantified by pixel number (u, v), where u and v are discrete integer values representing coordinates along the *x*- and *y*- direction on the photosensor plane. This results in discrete values of depth and hence limited accuracy. To overcome this limitation, the SAD values for 4-neighbourhoods of the best-matched vector are taken into consideration. The refined value of translation vector is $(u + \Delta u, v + \Delta v)$, where Δu and Δv are given by:

$$\Delta x = 0.5 \times \frac{(SAD(u-1,v)-SAD(u,v))-(SAD(u+1,v)-SAD(u,v))}{(SAD(u-1,v)-SAD(u,v))+(SAD(u+1,v)-SAD(u,v))} \quad \text{(S10)}$$

$$\Delta y = 0.5 \times \frac{(SAD(u,v-1)-SAD(u,v))-(SAD(u,v+1)-SAD(u,v))}{(SAD(u,v-1)-SAD(u,v))+(SAD(u,v+1)-SAD(u,v))} \quad \text{(S11)}$$

where $SAD(u, v)$ is the SAD value for the best-matched translation vector, $SAD(u-1, v)$, $SAD(u, v)$ and $SAD(u, v)$, $SAD(u, v)$ are the SAD values for 4-neighbourhoods of the best-matched vector. The refined value of the translation vector is shifted towards the neighbourhood with a lower SAD value, with Δu and Δv both below 0.5 pixels. In this way, we obtain successive depth values and, consequently, higher depth estimation accuracy. The shift of the rotation angle due to off-axis aberration[5] is also corrected.

To further verify the robustness of the 4D imaging retrieval framework, we performed imaging experiments on alternative scene with up to 8 objects, with results shown in **Fig. S11**. The mean error of depth estimation is still well below 1% (**Fig. 3f** in the main text).

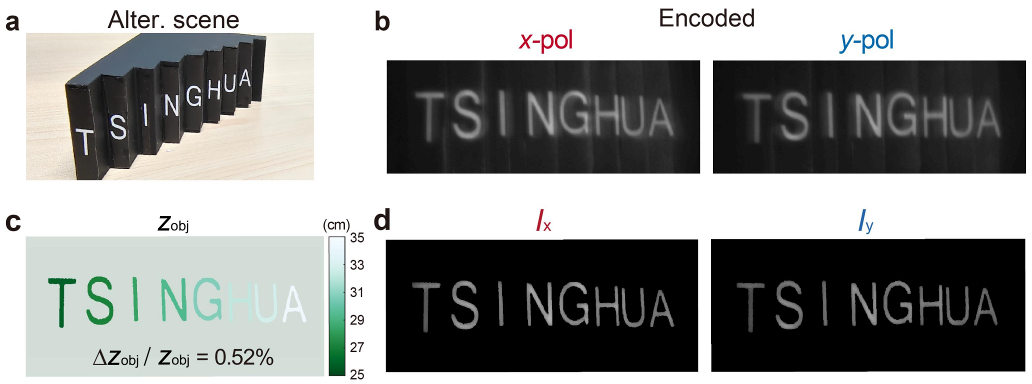

Figure S11 | a, Photograph of the alternative scene. **b**, Encoded image-pair captured by the metasurface camera. **c**, Retrieved depth map of the alternative scene, with a normalized mean absolute error ($NMAE$) of 0.52%, respectively. **d**, Decoded all-in-focus polarization image pair.

We also quantitatively evaluate the depth estimation error for outdoor dynamic scene, as is shown in **Fig. S12**. The NMAE of depth estimation for the still and the moving toy car are 0.78% and 1.26%, respectively. The slightly higher depth estimation error for the outdoor dynamic scene, in comparison with indoor static scenes, may be due to the longer depth range, motion artifact and additional noise.

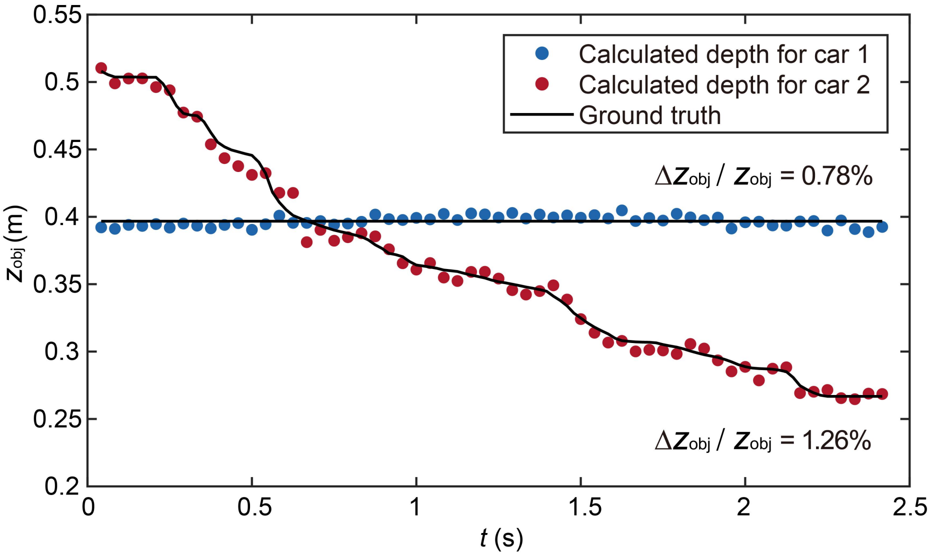

Figure S12 | Comparison of the calculated depth to the ground truth as a function of time, for the outdoor dynamic scene shown in Figure 4 of the main text.

6. Depth estimation range and accuracy

At a given z_{obj}, the mean absolute error (MAE) of the estimated depth is inversely proportional to the rotation speed of the single-helix PSF. According to Supplementary Section 1, the rotation speed of the single-helix PSF,

$$\frac{\partial \varphi}{\partial z_{\mathrm{obj}}} \propto \frac{\partial \zeta}{\partial z_{\mathrm{obj}}} = -\frac{\pi}{\lambda} \cdot \frac{D^2}{4} \cdot \frac{1}{z_{\mathrm{obj}}^2} \quad \text{(S12)}$$

where $D = 2R$ is the diameter of the metasurface aperture. The rotation speed of the single-helix PSF as a function of z_{obj} and D is shown in **Fig. S13**. According to Eq. S12, MAE of the estimated depth is inversely proportional to the square of D and proportional to the square of z_{obj}. The normalized mean absolute error ($NMAE$, defined as MAE / z_{obj}) of the estimated depth is also inversely proportional to the square of D but proportional to z_{obj}. Therefore, a metasurface with a larger aperture diameter can perform equally high accuracy depth estimation (with the same $NMAE$) for much further distances. A metasurface with a 5-cm-diameter can have an $NMAE \leq 1\%$ for depth estimation at a 200-m-range.

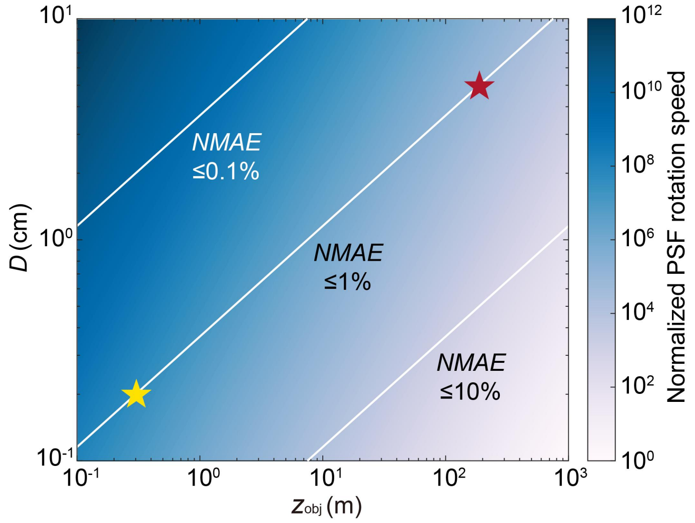

Figure S13 | PSF rotation speed as a function of the axial depth of the object z_{obj} and the diameter of the metasurface aperture D. The white lines are contours of equal relative depth estimation accuracy. The yellow star corresponds to the demonstrated prototype metasurface camera with $NMAE \leq 1\%$ at $D = 0.2$ cm, $z_{\mathrm{obj}} = 0.3$ m. The red star indicates a metasurface with $D = 5$ cm can have $NMAE \leq 1\%$ at an axial depth of 200 m.

7. Depth estimation accuracy quantified by the Fisher information

To evaluate the depth estimation accuracy of an imaging system with single-helix PSFs, we use a statistical information theory measure called the Fisher information[7,10,11]. Fisher information measures the sensitivity of the PSF in response to changes of the 3D location of the scene point under the inevitable existence of noise. The Fisher information is given by:

$$FI_z(\mathbf{r}) = \frac{1}{\sigma_N^2} \sum_{u=1}^{N_u} \sum_{v=1}^{N_v} \left(\frac{\partial PSF(u,v;\mathbf{r})}{\partial z} \right)^2 \quad \text{(S13)}$$

where $\vec{r} = (x, y, z)$ is the 3D location of the target object. $FI_z(\vec{r})$ is the Fisher information of the PSF along the depth (z) dimension. u and v are the coordinates of the pixel on the photosensor plane, with a total number of N_u and N_v along each direction. $PSF(u, v; \vec{r})$ is

the PSF's intensity value at the pixel (u, v) for the target object at $\vec{r}$, which is normalized such that the maximum irradiance is equal to one. σ_N^2 is the variance of per-pixel noise. The reciprocal root of Fisher information is the Cramér-Rao lower bound (CRLB), which is the lower bound of the depth estimation variance over all unbiased estimators given by:

$$E(z_e - z)^2 \geq \sigma_{\mathrm{CRLB}}^2 = [FI_z(\mathbf{r})]^{-1} \quad \text{(S14)}$$

where z_e is the estimated depth of the target object. Therefore, σ_{CRLB} quantifies the theoretical limit of the depth estimation accuracy. We calculate the σ_{CRLB} for depth estimation using a standard lens and an optimized pair of single-helix PSFs, respectively, with the results shown in **Fig. S14**. Both imaging systems are assumed to have an aperture size and focal length identical to the prototype metasurface camera. The signal-to-noise ratio is assumed as 10, resulting in $\sigma_{\mathrm{N}} = 0.1$ for each imaging system.

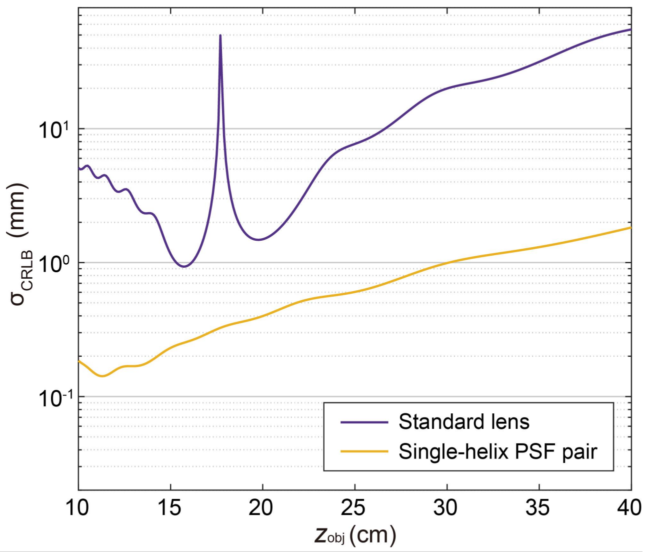

Figure S14 | Calculated standard deviation of the lower limit of depth estimation accuracy σ_{CRLB} for the PSF of a standard lens and an optimized pair of single-helix PSFs, respectively.

As is shown in **Fig. S14**, an imaging system with a pair of single-helix PSFs has a theoretical limit of depth estimation variance that is over two orders of magnitude less than an imaging system equipped with a standard lens.

8. Extended depth of field imaging

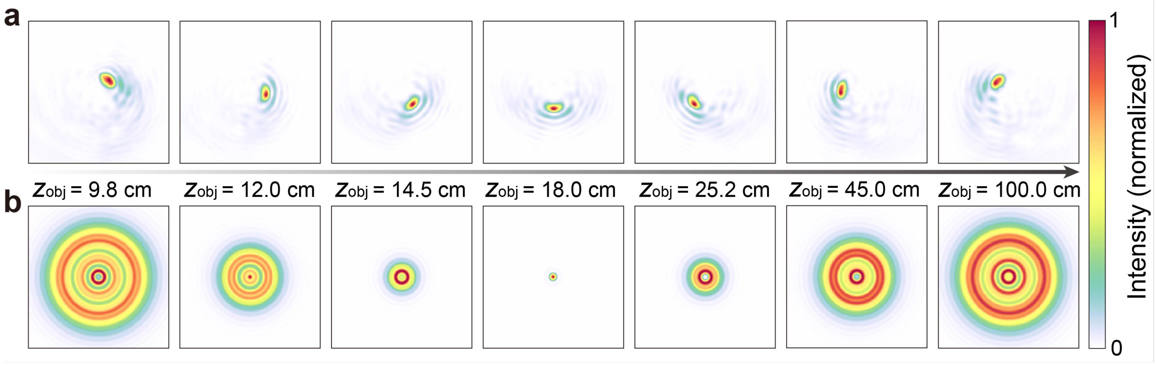

Figure S15 | a, Calculated PSFs of the metasurface for point light sources at different axial depths, for *x*-polarized incident light. **b,** Calculated PSFs of a standard lens for point light sources at different axial depths.

The metasurface lens based on the single-helix PSF pair can acquire high-quality all-in-focus 2D images with an extended depth of field, as can be observed from the comparison between numerically calculated PSFs of the metasurface and a standard lens with identical aperture size and focal length for point light sources located at different axial depths, with the in-focus object plane both placed at z_{obj} = 18.0 cm. As is shown in **Fig. S15**, the PSFs of the metasurface can remain tight over a large depth range, while PSFs of the standard lens quickly become blurry.

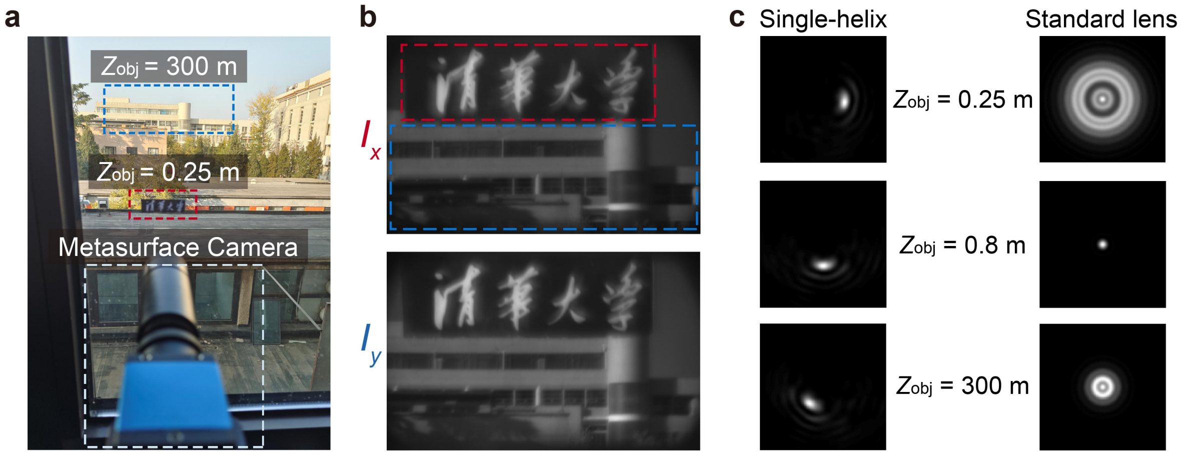

Figure S16 | a, Photograph of the metasurface camera and a scene composed of target objects located both at very far and close distances, respectively. **b**, Polarization image pair captured by the metasurface camera. **c**, Calculated PSFs of the metasurface and a standard lens, respectively, both with the in-focus object plane at z_{obj} = 0.8 m.

We further experimentally demonstrate the use of the metasurface camera for imaging over an extended depth of field. As is shown in **Fig. S16a,b**, images taken by the metasurface can remain sharp for both objects at very far and close distances (300 m and 0.25 m), respectively. In comparison, for a standard lens with the same aperture size and focal length, one of the

objects at a very far and close distance will inevitably become blurry due to the rapid spread of the PSF as a function of the object depth (**Fig. S16c**).

9. Design for full-Stokes polarization and depth imaging

As schematically shown in **Fig. S17a**, a relatively straightforward way to realize single-shot depth and full-Stokes polarization imaging is to use three groups of spatially multiplexed meta-atoms to independently control the light with three pairs of orthogonal polarization states. When imaging using such a metasurface, full-Stokes polarization and depth information of the target scene are encoded in six sub-images, corresponding to light of three pairs of orthogonal polarization states, on different areas of the photosensor plane, as shown in **Fig. S17b**. The PSFs of the six sub-images are three pairs of conjugate single-helix PSFs, as shown in **Fig. S17c**. Compared with the current design, such a design may further increase the accuracy and robustness of depth estimation since there are three pairs of sub-images with conjugate single-helix PSFs. The trade-off is that the pixel number of each output image is reduced by three times, if the total pixel number of the photosensor is fixed.

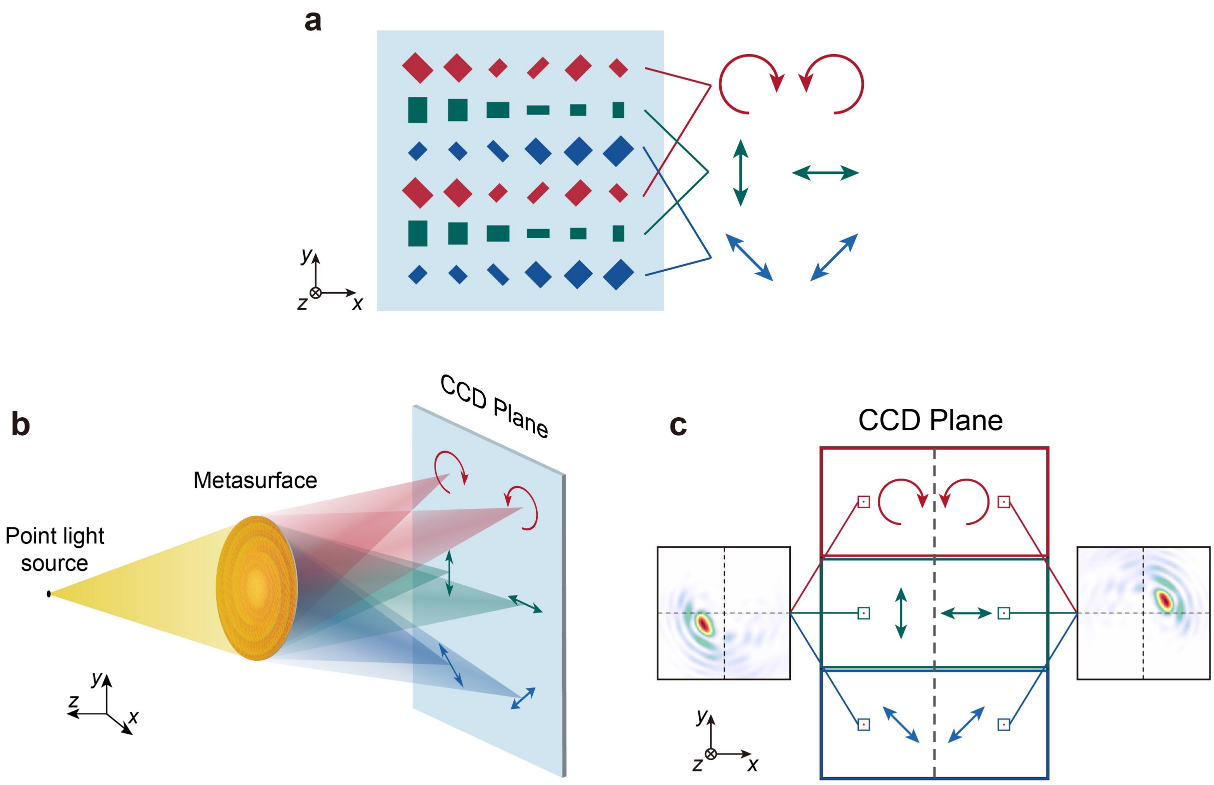

Figure S17 | a, Schematic of spatially multiplexed metasurface that can independently control light with 3 pairs of orthogonal polarization states, including 0° and 90° linear polarization, 45° and 135° linear polarization, and left- and right-circular polarization, respectively. **b,** Schematic of using a metasurface to image a point light source, with full-Stokes polarization and depth information of the point light source encoded in six sub-images, corresponding to 3 pairs of orthogonal polarization states, on different areas of the photosensor plane. **c,** Schematic

of 3 pairs of conjugate single-helix PSFs recorded on the photosensor plane, with each pair of orthogonal polarization states corresponding to a pair of conjugate single-helix PSFs.

References

1 Schechner, Y. Y., Piestun, R. & Shamir, J. Wave propagation with rotating intensity distributions. *Phys. Rev. E* **54**, R50-R53 (1996).

2 Montgomery, W. D. Self-Imaging Objects of Infinite Aperture. *J. Opt. Soc. Am.* **57**, 772-778 (1967).

3 Pavani, S. R. P. & Piestun, R. High-efficiency rotating point spread functions. *Opt. Express* **16**, 3484-3489 (2008).

4 Jin, C. *et al.* Dielectric metasurfaces for distance measurements and three-dimensional imaging. *Adv. Photonics* **1**, 036001 (2019).

5 Colburn, S. & Majumdar, A. Metasurface Generation of Paired Accelerating and Rotating Optical Beams for Passive Ranging and Scene Reconstruction. *ACS Photonics* **7**, 1529-1536 (2020).

6 Prasad, S. Rotating point spread function via pupil-phase engineering. *Opt. Lett.* **38**, 585-587 (2013).

7 Berlich, R. & Stallinga, S. High-order-helix point spread functions for monocular three-dimensional imaging with superior aberration robustness. *Opt. Express* **26**, 4873-4891 (2018).

8 Matsushima, K., Schimmel, H. & Wyrowski, F. Fast calculation method for optical diffraction on tilted planes by use of the angular spectrum of plane waves. *J. Opt. Soc. Am. A* **20**, 1755-1762 (2003).

9 John, C. A Computational Approach to Edge Detection. *IEEE Trans. Pattern Anal. Machine Intel.* **8**, 679-698 (1986).

10 Greengard, A., Schechner, Y. Y. & Piestun, R. Depth from diffracted rotation. *Opt. Lett.* **31**, 181-183 (2006).

11 Pavani, S. R. P., Greengard, A. & Piestun, R. Three-dimensional localization with nanometer accuracy using a detector-limited double-helix point spread function system. *Appl. Phys. Lett.* **95**, 021103 (2009).
