# Supplementary material for: Monocular metasurface camera for passive single-shot 4D imaging
Source: Nat Commun. 2023 Feb 23;14:1035. doi: 10.1038/s41467-023-36812-6 (PMC9950364; doi:10.1038/s41467-023-36812-6)
Supplement: Supplementary file 2 — Description of Additional Supplementary Files [file 41467_2023_36812_MOESM2_ESM.pdf]

Description of Additional Supplementary Files

Supplementary Movie 1: Movie of the imaging results of an outdoor dynamic scene corresponding to Figure 4 in the main text.
